# Nanohydroxyapatite/polyamide 66 strut subsidence after one-level corpectomy: underlying mechanism and effect on cervical neurological function

**DOI:** 10.1038/s41598-018-30678-1

**Published:** 2018-08-14

**Authors:** Weiyang Zhong, Xinjie Liang, Ke Tang, Xiaoji Luo, Zhengxue Quan, Dianming Jiang

**Affiliations:** 1 Department of Orthopedic Surgery, the First Affiliated Hospital of Chongqing Medical University, 400016 Chongqing, China; 2 Department of Pain Management, The First Affiliated Hospital of Chongqing Medical University, 400016 Chongqing, China

## Abstract

The aim of this study was to investigate n-HA/PA66 strut subsidence after one-level in Anterior cervical corpectomy decompression and fusion (ACCF) and its effect on treatment outcomes to better understand the underlying mechanism and related risk factors. In total, 56 patients undergoing ACCF using n-HA/PA66 struts were analysed retrospectively. After a 12-month follow-up, the height of the fused segments and fused intervertebral heights were measured, the neurological findings were evaluated using the Japanese Orthopedic Association (JOA) and axial pain was assessed using a Visual Analogue Scale(VAS). Subsidence was defined as a decrease in the height of the fused segments or the fused intervertebral body greater than 3 mm compared with that on postoperative day one, and all patients were assigned to the n-HA/PA66 strut subsidence and control groups. In total, 45 patients experienced n-HA/PA66 strut subsidence during the postoperative (3 ± 2.42/3.11 ± 2.01) months. No significant differences were observed in sex, age, hospitalization time, surgical haemorrhage,bone mineral density (BMD), or height in the n-HA/PA66 strut group. The JOA and VAS of neck pain in the control group improved more than those in the subsidence group, suggesting that subsidence might be correlated with poor improvement of neurological function. In conclusion, n-HA/PA66 strut subsidence is a common complication after ACCF, and the reduced height of the postoperative fused segments and the height reduction in the postoperative fused intervertebral bodies are independent risk factors of n-HA/PA66 strut subsidence.

## Introduction

ACCF is considered a safe and effective surgical procedure for the treatment of more than one level of cervical degenerative lesions, particularly multiple cervical spondylotic myelopathy(CSM), while providing direct decompression, maintaining cervical involvement and providing the adjacent segment stability^1–3^. ACCF is widely used by spine surgeons, and its excellent surgical outcomes have been confirmed several studies^1–8^. Compared with the traditional iliac bone transplant, a titanium mesh cage (TMC)^4–6^ using an n-HA/PA66 strut with a locking titanium plate can reduce long-term complications in the bone graft area and complications of TMC subsidence. The n-HA/PA66 strut is a novel non-metal implant composed of nanohydroxyapatite and polyamide 66^9,10^, whose specific proportions simulate natural bones, and has been applied clinically for more than ten years in China. The safety and mechanical particularities of the n-HA/PA66 strut have been documented in previous studies. Due to the popularity of the n-HA/PA66 strut, surgeons have gradually noted that the drawbacks, such as long-term bony fusion and subsidence. Recently, the incidence of n-HA/PA66 strut subsidence which could lead to postoperative yellow ligament folds, cervical kyphosis, and neural foramen stenosis, was reported to be 2.86–19.68%^7,8,11,12^.

To date, no study has investigated n-HA/PA66 strut and its potential risk factors of subsidence in one-level corpectomy in ACCF. The purpose of this study was to investigate the factors related to n-HA/PA66 strut subsidence and its effect on cervical neurological function.

## Materials and Methods

### Patient population

This retrospective study was approved by the Institutional Review Board of the First Affiliated Hospital of Chongqing Medical University. The methods used in this study were conducted according to the approved guidelines, and informed consent was obtained from all patients. The n-HA/PA66 strut was designed and fabricated by the Institute ofMaterials Science and Technology, Sichuan University, and our department and was approved for clinical use in 2005 by the State Drug and Food Administration of China. Between January and June of 2016, 56 patients (21 men and 35 women) with CSM who underwent one-level corpectomy with a n-HA/PA66 strut and a locking titanium plate at our department performed by a spine senior surgeon, were retrospectively investigated. All patients manifested disturbances associated with myelopathy, which was verified by radiographic data, including X-ray, magnetic resonance images (MRI), and computed tomography (CT) as necessary.

### Surgical technique

**Figure 1:**
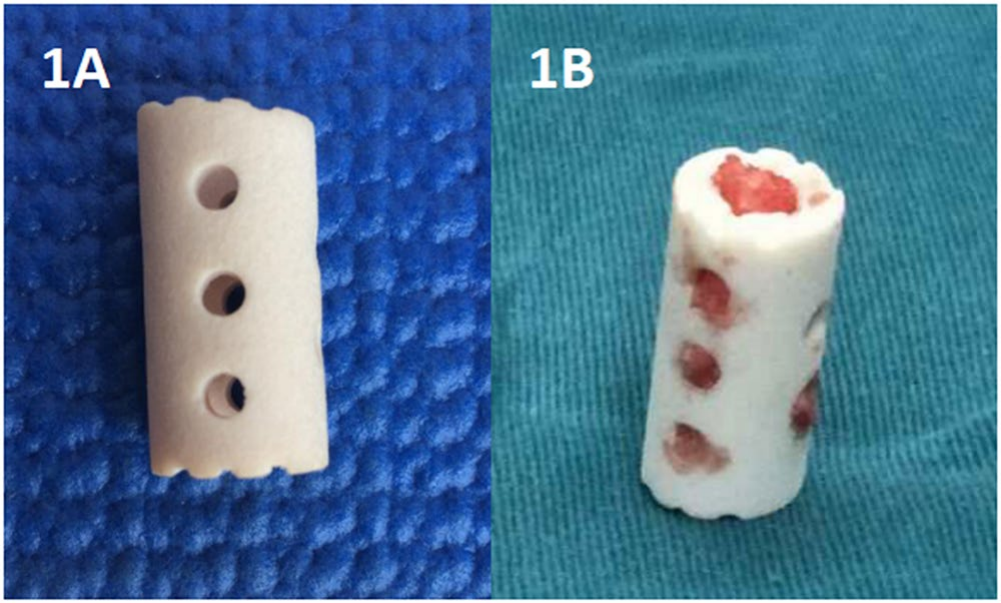
Lateral and frontal view photographs of a nanohydroxyapatite/polyamide 66 strut.

All surgeries were performed by the same senior spine surgeon. Under general anaesthesia, the neck was slightly extended, and the cartilage endplates were carefully removed using curettes,while the bony endplates were preserved. An n-HA/PA66 strut filled with cancellous bone from the resected vertebral body was applied to enhance the contact areas in each case. An n-HA/PA66 strut (Fig. 1) was placed into the intervertebral space under light distraction.Finally, the anterior cervical plate (Johnson and Johnson Co. Depuy Spine Ltd., Raynham, MA, U.S.A.) was fixed across the fused segments. Surgical haemorrhage and the operation time were recorded. Postoperatively, all patients were required to wear a Philadelphia hard cervical collar for six to eight weeks. In addition, the patients began training their neck muscles step-by-step.

### Outcome assessment

All patients underwent radiographs of the cervical spine 1 day, 3 months, 6 months, and 12 months postoperation, and the fused segmental height (the distance between the midpoint of the adjacent vertebrae) and the fused intervertebral height (the distance between the midpoint of the inferior endplate of the upper vertebral body and the midpoint of the superior endplate of the lower vertebral body) were measured. The measurements obtained 3, 6 and 12 months after the operation were compared with those obtained 1 day after the operation. n-HA/PA66 strut subsidence was defined as a decrease in the fused segmental height or fused intervertebral height during the follow-up period greater than 3 mm compared with that on day 1, and the patients were divided into the subsidence group and the control group. All radiographic data and measurements in this study were reviewed by two senior spine surgeons and one senior radiologist. In addition, the neurological statuses of the patients were assessed using the JOA score before and 12 months after the operation, and the improvement rate was calculated. The VAS of neck pain was evaluated. According to the Cobb method, cervical lordosis was defined as the angle formed between the inferior endplates of C2 and C7, and the angle of the aligned fused segments was defined as the angle formed between the superior endplate of the upper vertebral body and the inferior endplate of the lower vertebral body. The segmental angle was assessed by the angle of the fused vertebra.

### Statistical analysis

The statistical analysis was performed using Student’s t-test, and a correlation analysis was performed for multiple comparisons using Statistic Analysis System (SAS Institute Inc., Cary, NC, USA). The results are expressed as the group means ± SD. Differences with a P-value < 0.05 were considered significant.

## Results

**Figure 2:**
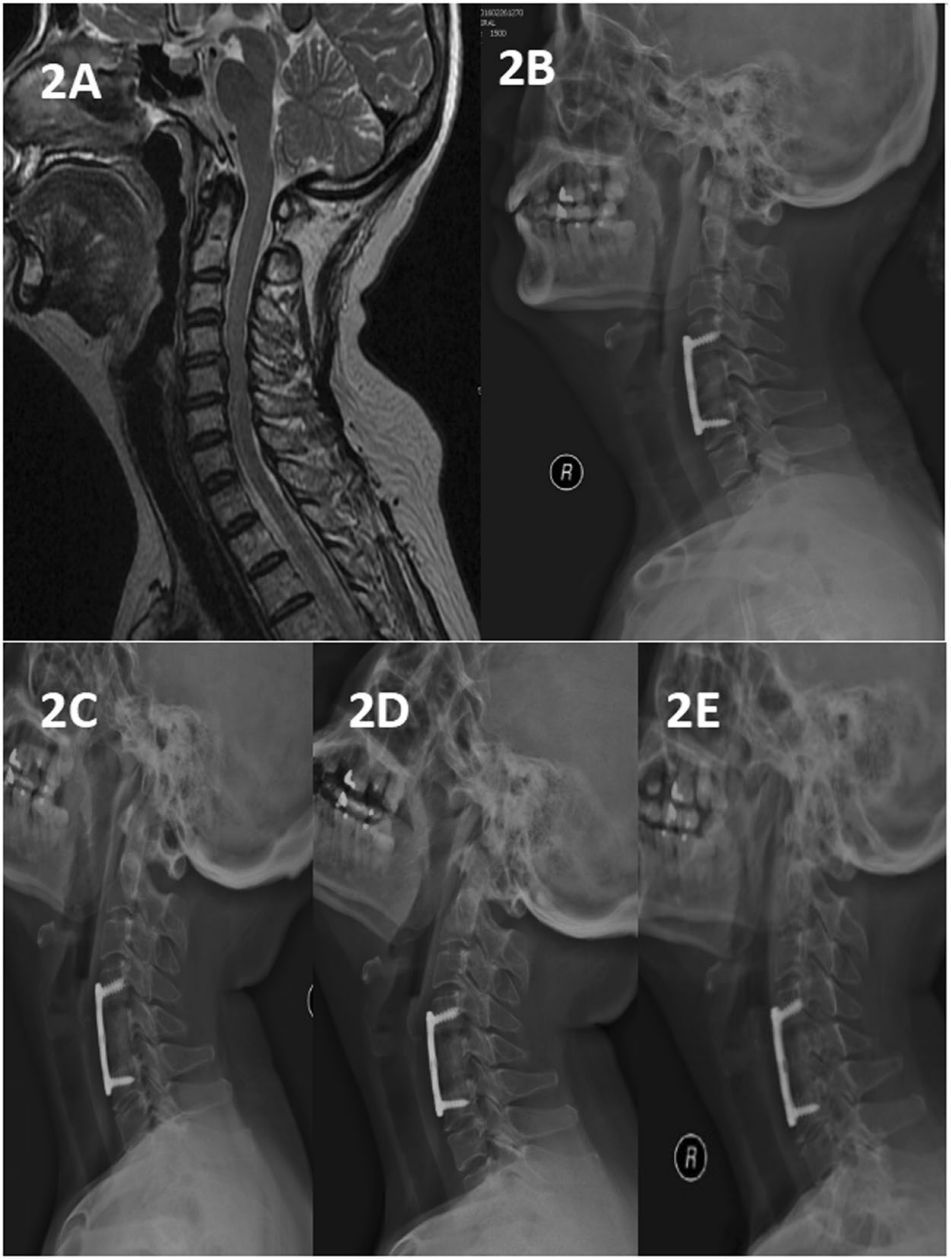
A 50-year-old woman who underwent 1-level corpectomy with a nanohydroxyapatite/polyamide66 strut for cervical reconstruction. The preoperative cervical MRI (**A**) revealed C4/5 disc herniation. The patient underwent a C5 corpectomy and fusion using an n-HA/PA66 strut (**B**). The lateral X-ray film indicated that the autogenous bone granules filling the strut had achieved bony fusion, and subsidence was observed at the 3-month follow-up (**C**). The strut and internal fixation were in position at 3, 6, and 12 months of follow-up (**C**–**E**).

**Figure 3:**
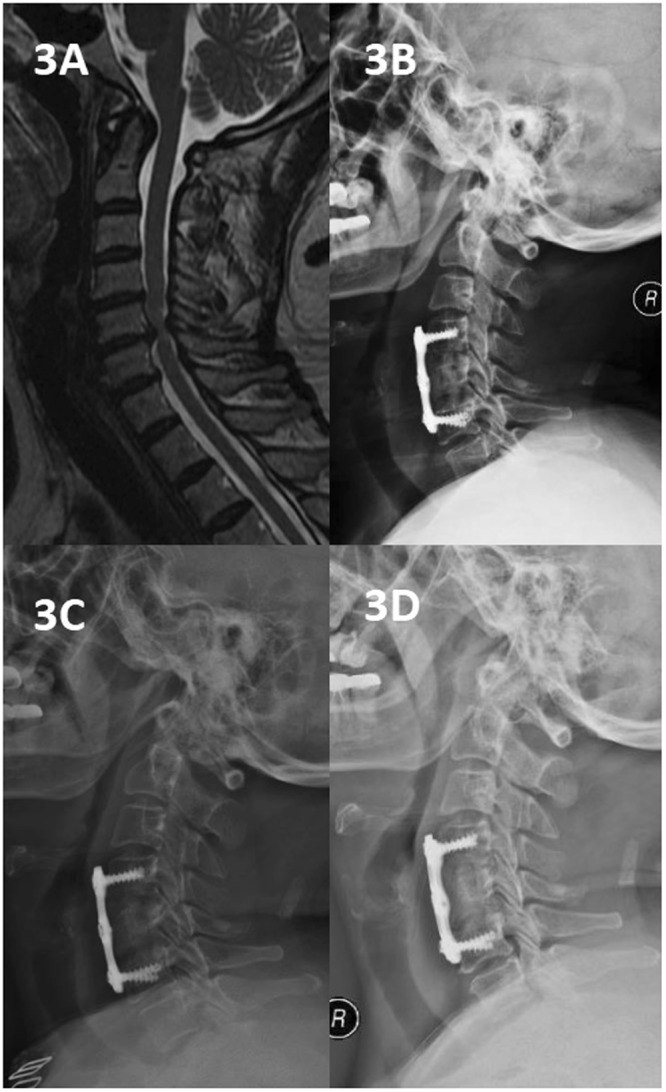
A 60-year-old woman who underwent 1-level corpectomy with a nanohydroxyapatite/polyamide66 strut for cervical reconstruction. The preoperative cervical MRI (**A**) revealed C5/6 disc herniation. The patient underwent a C5 corpectomy and fusion using an n-HA/PA66 strut (**B**). The lateral X-ray film indicated that the autogenous bone granules filling the strut had achieved bony fusion, and subsidence was observed at the 6-month follow-up (**C**). The strut and internal fixation were in position at 3, 6, and 12 months of follow-up (**B**–**D**).

**Figure 4:**
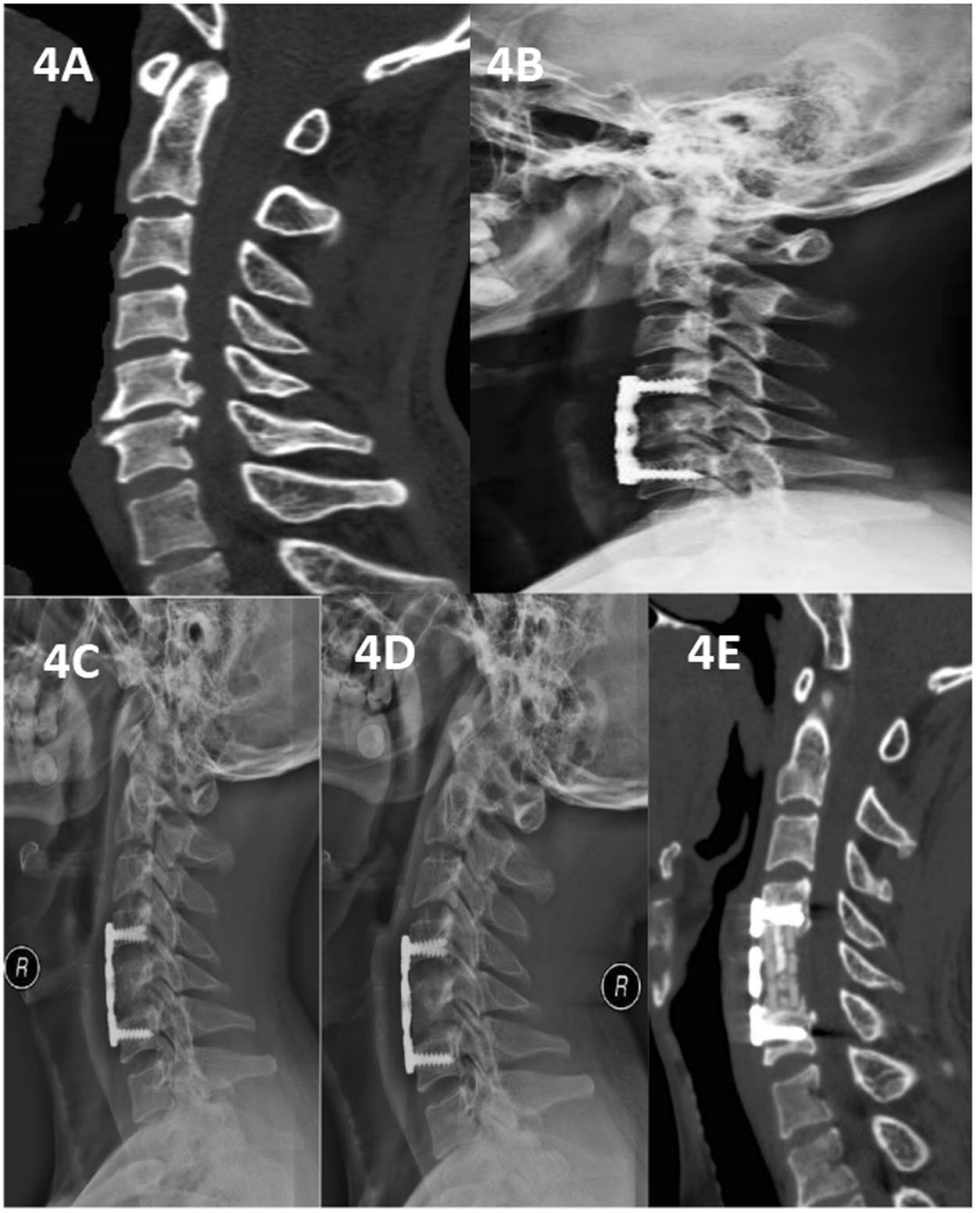
A 55-year-old man who underwent 1-level corpectomy with a nanohydroxyapatite/polyamide66 strut for cervical reconstruction. The preoperative cervical CT (**A**) revealed C5/6 disc herniation and osteophyte proliferation. The patient underwent a C5 corpectomy and fusion using an n-HA/PA66 strut (**B**). The lateral X-ray film and CT indicated that the autogenous bone granules filling the strut had achieved bony fusion, and subsidence was observed at the 6-month follow-up (**C**,**E**). The strut and internal fixation were in position at 3, 6, and 12 months of follow-up (**B**–**D**).

In total, 56 patients were followed-up for 12 months, and the results were analysed. In total, 45 patients developed postoperative n-HA/PA66 strut subsidence, and the incidence rate was 80% (45/56). The average occurrence time postoperatively was 3 ± 2.42/3.11 ± 2.01 months (Figs 2, 3 and 4), and occurrence was defined as a decrease in the fused segmental height and fused intervertebral height greater than 3 mm.

To investigate the potential risk factors of postoperative n-HA/PA66 strut subsidence, general (age, sex, hospital stay, etc.) and operation-related information of the two groups was statistically analysed. No significant differences were observed in sex, age, hospitalization time, surgical haemorrhage, BMD, height of n-HA/PA66 strut, or fusion rate between the two groups (P = 0.7015, 0.7028, 0.7326, 0.7107, 0.3417, 0.5024, 0.1972, 0.2541), except for drainage (P = 0.7015), which was slightly reduced in the control group (Table 1).

**Table 1:** Comparison of the baseline and postoperative data.

	Control group	Subsidence group	p
No. of patients (n)	11	45	
Males/females (n)	7/4	18/27	0.7015
Mean age	56.36 ± 7.13	55.36 ± 14.06	0.7028
Hospitalization time (days)	11.11 ± 1.78	10.72 ± 1.62	0.7326
Surgery time (minutes)	108.42 ± 20.16	106.00 ± 12.10	0.7107
Surgical haemorrhage (ml)	114.54 ± 104.53	124.44 ± 130.83	0.3417
Drainage (ml)	15.27 ± 5.08	17.69 ± 9.80	0.0301
BMD (L/H)	−2.20 ± 0.70/−2.28 ± 0.67	−2.23 ± 0.43/−2.19 ± 0.44	0.5024
Height of n-HA/PA66 strut (mm)	23.72 ± 1.90	22.78 ± 2.45	0.1972
Fusion rate (12 months post-op)	90.90% (10/11)	91.11% (41/45)	0.2541

The radiographic and clinical outcomes of the two groups were statistically analysed. The reduced height of the postoperative fused segments, reduced height of the postoperative fused intervertebral bodies, height loss of the fused segments, height loss of the fused intervertebral bodies, reduced angle of the cervical alignment, and reduced angle of the fused segment alignment significantly differed (P < 0.0001) (Table 2).

**Table 2:** Radiographic and clinical outcomes in each group.

Parameter	Control group	Subsidence group	P
Reduced height of postoperative fused segments	2.46 ± 2.87	7.94 ± 4.85	<0.0001
Reduced height of postoperative fused intervertebral body	4.36 ± 3.61	6.92 ± 3.56	<0.0001
Height loss of fused segments (mm)			
1 month post-op	0.91 ± 0.52	2.91 ± 1.67	<0.0001
3 months post-op	1.40 ± 0.52	5.25 ± 2.90	<0.0001
6 months post-op	1.78 ± 0.54	5.93 ± 2.81	<0.0001
12 months post-op	2.15 ± 0.48	6.62 ± 2.92	<0.0001
Height loss of fused intervertebral body (mm)			
1 month post-op	0.88 ± 0.53	2.38 ± 1.09	<0.0001
3 months post-op	1.77 ± 0.46	4.34 ± 2.01	<0.0001
6 months post-op	2.10 ± 0.44	5.08 ± 2.07	<0.0001
12 months post-op	2.38 ± 0.35	5.54 ± 1.82	<0.0001
Reduced Angle of Cervical alignment (°)	−1.30 ± 14.92	2.05 ± 10.38	<0.0001
1 month post-op	−2.33 ± 16.57	2.85 ± 9.68	<0.0001
3 months post-op	0.57 ± 14.55	4.83 ± 9.81	<0.0001
6 months post-op	1.47 ± 15.70	5.99 ± 9.28	<0.0001
12 months post-op	21.06 ± 55.69	8.47 ± 9.08	<0.0001
Reduced Angle of fused segments alignment (°)	2.70 ± 11.13	1.92 ± 8.15	<0.0001
1 month post-op	4.11 ± 10.85	1.54 ± 3.61	<0.0001
3 months post-op	5.61 ± 10.66	2.57 ± 5.13	<0.0001
6 months post-op	5.16 ± 10.67	3.26 ± 5.99	<0.0001
12 months post-op	6.12 ± 10.78	5.97 ± 5.45	<0.0001
VAS of neck pain (points)			
Pre-op	6.73 ± 0.65	6.64 ± 0.65	0.0591
Final follow-up	1.67 ± 0.64	2.73 ± 0.50	0.0309
JOA score (points)			
Pre-op	9.64 ± 1.36	9.36 ± 1.13	0.3072
Final follow-up	15.9 ± 0.94	14.42 ± 0.58	0.0347

The JOA and VAS of neck pain in the control group were higher than those in the subsidence group (P = 0.0347, 0.0309), suggesting that n-HA/PA66 strut subsidence might be correlated with poor improvement in neurological function after surgery.

In the subsidence group, a correlation was observed between the height loss of the fused segments and the height loss of the fused intervertebral bodies and their potential risks. The height loss of the fused segments was correlated with a reduced height of the postoperative fused segments (r = 0.51191, P = 0.0003), while the height loss of the fused intervertebral bodies was correlated with a reduced height of the postoperative fused intervertebral bodies (r = 0.47167, P = 0.0011); however, no correlation was observed with age (r = 0.2195, P = 0.1474), sex (r = 0.0.09305, P = 0.5432), BMD (r = 0.08545, P = 0.1236), etc.

Outcome and complications: No cerebrospinal fluid leakage, incisional wound infection, or wound haematoma was observed in either group. However, axial pain in the neck was more commonly found in the patients in the subsidence group (36/45 vs. 4/11, P < 0.001).

## Discussion

The measurement of the intervertebral height and diagnostic criteria for n-HA/PA66 strut subsidence have been controversial^7,8,11,12^. The fused segmental height (the distance between the midpoint of the adjacent vertebrae) was measured, and any distance >2 mm or >3 mm was considered n-HA/PA66 strut subsidence. Differences in the cervical curvature resulting in uncertain poor-quality data may affect the diagnosis of postoperative n-HA/PA66 strut subsidence. In our study, the intervertebral body height (the distance between the excised vertebrae) was defined as the distance between the midpoint of the inferior endplate of the upper vertebral body and the midpoint of the superior endplate of the lower vertebral body. In addition, n-HA/PA66 strut subsidence was defined as no subsidence (intervertebral height loss = 0), mild subsidence (intervertebral height loss <3 mm) and severe subsidence (intervertebral height loss >3 mm). Due to the magnification of the X-ray, the measurement was greater than 3 mm, which was more accurate. In this study, n-HA/PA66 strut subsidence was defined as a decrease in the intervertebral height greater than 3 mm and a fused segmental height >3 mm. In our study, the two diagnostic criteria were correlated (r = 0.0.75978, P < 0.001). Therefore, we performed two measurements to evaluate subsidence, and the incidence rate was 80% (45/56), which was higher than that reported in previous studies. These results emphasize the need to strictly observe patients during follow-up.

The risk factors of TMC subsidence have been well documented^6,8,13–18^, but no study has reported the potential risk factors of subsidence following the use of an n-HA/PA66 strut in one-level corpectomy for ACCF. Regarding the biomechanics and considering safety, the pressure of an n-HA/PA66 strut for ACCF on both endplates of the adjacent vertebral bodies, is greater than that on the non-surgical segment endplates, which could be a direct cause of n-HA/PA66 strut subsidence. In addition, during the operation, excessive intervertebral distraction can result in stress concentration and subsidence of the n-HA/PA66 strut, which was confirmed in our study. The height loss of the fused segments was correlated with the reduced height of the postoperative fused segments (r = 0.51191, P = 0.0003), while the height loss of the fused intervertebral body was correlated with the reduced height of the postoperative fused intervertebral body (r = 0.47167, P = 0.0011). However, intervertebral distraction helps enlarge the neural foramen to improve the clinical effect. Future studies should investigate methods that avoid over-distraction or control distraction in detail. Although surgical procedures and thorough cleaning are performed cautiously, damage to the endplates and firm fixation should be strictly avoided.We also found that the average occurrence time of subsidence postoperatively was 3 ± 2.42/3.11 ± 2.01 months.Probably the subsidence occurred in the process of strut bone fusion and reconstruction.Animal research of bone-strut bony fusion interface are needed to analyze.

Several previous studies^6,8,13,14,19^ have demonstrated the risk factors of n-HA/PA66 strut subsidence, including age and sex, which are often used to predict subsidence. Due to the decline in BMD and poor bone quality, elderly patients and women (particularly postmenopausal women) are considered more prone to subsidence of the n-HA/PA66 strut. However, according to our study, there is no significant difference in age, sex and BMD between the patients in the subsidence group and the patients in the non-subsidence group, and subsidence was not correlated with age (r = 0.2195, P = 0.1474), sex (r = 0.0.09305, P = 0.5432), and BMD (r = 0.08545, P = 0.1236).

In the subsidence group, the JOA and VAS of neck pain were higher than those in the n-HA/PA66 strut non-subsidence group (P = 0.0347, 0.0309); however, subsidence did not lead to significant clinical symptoms or signs or severe kyphosis. However, we selected only patients with one-level corpectomy. Nonetheless, axial pain, which was principally caused by the ligamentum flavum wrinkle and the neural foramen stenosis following subsidence, was more frequently observed in the subsidence patients. When subsidence results in recompression of the spinal cord and nerve roots, neurological function might deteriorate.

## Conclusion

In summary, the observed incidence rate of subsidence was higher than expected using n-HA/PA66 struts for cervical one-level corpectomy. However, two factors were responsible for this higher incidence, i.e., over-reduced height of the postoperative fused segments and fused intervertebral body. Before further applications of n-HA/PA66 struts, the n-HA/PA66 strut height should be optimized, and more detailed measurements of the distraction should be performed. In addition, improved clinical outcomes could be observed. This study was a small-sample retrospective study, and prospective, randomized studies with long-term follow-up periods are needed.
